# Examination of parotid gland in diabetics with ultrasound elastography and microvascular imaging

**DOI:** 10.4317/medoral.26865

**Published:** 2024-10-13

**Authors:** Sule Erdem, Alptekin Tosun

**Affiliations:** 1 Assistant Professor, DDS. Department of Dentomaxillofacial Radiology, Faculty of Dentistry, University of Giresun, Giresun, Turkey; 2 Professor, MD. Department of Radiology, Faculty of Medicine, University of Giresun, Giresun, Turkey

## Abstract

**Background:**

Diabetes mellitus is a prevalent metabolic disorder that can result in the non-enflamatuar enlargement of the parotid gland. It is recognised as a condition that can impair microvascular perfusion.

**Material and Methods:**

The parotid glands of 38 individuals with diabetes and 38 healthy controls were examined bilaterally using ultrasound to assess parameters of volume, stiffness and microvascularity.

**Results:**

A statistically significant increase in parotid gland volume and a statistically significant decrease in microvascularity were observed in diabetic patients. Furthermore, a statistically significant increase in parotid gland volume was noted in diabetic individuals who were using antidiabetic drugs in comparison to non-users. A significant negative correlation was identified between the duration of exposure to diabetes and microvascularity.

**Conclusions:**

Ultrasonographic imaging can be used to assess the dimensions and microvascularity of the parotid gland.

** Key words:**Ultrasound, parotid gland, diabetes mellitus, elastography, microvascular imaging.

## Introduction

Diabetes mellitus (DM) is a chronic metabolic disorder characterised by high blood glucose levels (1). Diabetes represents a significant global health concern, with a rising prevalence. According to the International Diabetes Federation, the global prevalence of diabetes was estimated to be 9.3% in 2019, affecting 463 million people, and is projected to increase to 10.2% (578 million) by 2030 and 10.9% (700 million) by 2045 (2). The primary causes of DM are autoimmune destruction of pancreatic β cells, which results in insufficient insulin production, and resistance of body cells to insulin action. Type 1 DM is characterised by insufficient insulin production and thus requires regular administration of insulin analogues. Type 2 DM, also known as non-insulin dependent diabetes, is typified by insulin resistance and may also manifest as insulin deficiency as the disease progresses (1). The chronic hyperglycaemia that results from diabetes can give rise to a number of macrovascular and microvascular complications, including ischaemic heart disease, stroke, neuropathy, nephropathy and retinopathy (3).

The diagnosis of DM encompasses a range of methods, including the oral glucose tolerance test, glycated hemoglobin (HbA1c) measurements, and genetic testing (4,5). HbA1c is a widely used biomarker for the diagnosis of DM and for assessing long-term glycemic control in individuals with DM. The American Diabetes Association recommends including HbA1c levels of 6.5% or higher as a criterion for diagnosing DM (6).

Sialodenosis (or sialosis) is a rare condition characterised by diffuse, non-inflammatory, non-neoplastic enlargement of the major salivary glands. Its aetiology is multifaceted and encompasses a range of systemic factors. It has been demonstrated that patients with diabetes may present with asymptomatic bilateral parotid gland sialodenosis, which may be attribuTable to a number of factors, including fatty infiltration and acinus hypertrophy. Sialadenosis in DM is characterised by the hypertrophy of the parotid acini, which typically measure 40 µm in diameter but can increase to as much as 100 µm, resulting in clinically visible glandular hypertrophy (7).

Imaging of the salivary glands is a fundamental component of the diagnostic process for a range of conditions affecting these structures. A variety of imaging modalities are employed for the assessment of the salivary glands, including sialography, ultrasonography, computed tomography (CT), magnetic resonance imaging (MRI), and salivary gland scintigraphy. These techniques provide detailed information about the anatomy, function, and pathology of the salivary glands (8). Ultrasonography is a valuable, non-invasive, and widely available tool for imaging the major salivary glands. It offers detailed information on the glandular parenchyma and aids in the early detection of abnormalities. Research has demonstrated that ultrasonography of the major salivary glands correlates positively with histological findings (9).

Ultrasound elastography is an imaging technique that is employed to assess tissue elasticity by measuring the stiffness of the tissue in question through the use of ultrasound imaging. This method, also referred to as virtual palpation, employs a range of techniques, including strain elastography (SE) and shear wave elastography (SWE), to assess tissue stiffness. Strain elastography, which assesses tissue stiffness without the need for invasive procedures, provides additional information about the mechanical properties of tissues by coding them in different colours according to their stiffness characteristics. This technique is useful for the diagnosis and characterisation of lesions (10).

Ultrasound microvascular imaging is a non-invasive processing technique based on Doppler ultrasound that allows for the assessment of microvascular distribution and perfusion. It has demonstrated superior sensitivity in detecting blood signals compared to conventional Doppler imaging. These techniques provide high-resolution images of microvessels, allowing detailed visualisation of low-flow vessels and improving the assessment of microvascular perfusion in various clinical conditions (11).

The objective of this study was to examine the volume, stiffness and microvascular perfusion properties of bilateral parotid glands in diabetic and healthy subjects using ultrasonography. To the best of our knowledge, this is the first study to utilise microvascular imaging to investigate the parotid glands of individuals with diabetes.

Materıal and Methods

- Study design and ethics

The study was designed as a prospective cross-sectional study and was conducted in accordance with the ethical principles set forth in the Declaration of Helsinki. Approval for the study was granted by the Clinical Research Ethics Committee (Decision number: 20.11.2023/01). All participants were informed about the study and provided written informed consent. A power analysis was conducted to determine the optimal sample size. Accordingly, the study population comprised 38 individuals with diabetes and 38 healthy controls, all aged 18 years or over. The diagnosis of patients who reported a history of diabetes was corroborated through an examination of their medical records. The HbA1c value and the duration of exposure to diabetes were obtained from the medical records and recorded on pre-prepared forms. In the absence of an HbA1c value recorded within the preceding three months in the medical records of participants with diabetes, they were excluded from the study. The exclusion criteria were as follows: major salivary gland surgery, any pathology such as a tumour, infection, etc. in major salivary glands, Sjogren's syndrome, chronic alcoholism, anorexia nervosa, connective tissue diseases such as scleroderma affecting skin elasticity, history of head and neck radiotherapy, cardiovascular disease, haematologic disease, pharmacologic agent use affecting blood flow, endocrine diseases other than DM.

- Imaging protocol

The parotid glands of the participants were imaged with an Esaote MyLab X7 ultrasound device in the department of dentomaxillofacial radiology. All imaging was conducted by a radiologist with 22 years of experience and no knowledge of the subjects' group allocation. Subjects were instructed to abstain from food and drink, as well as chewing gum, for a period of two hours prior to the imaging procedure. Subjects in which the deep lobe could not be visualised with sufficient clarity and in which part of the gland was hidden behind the ramus mandibula were excluded from the study. A L4-15 linear probe was employed, and the ultrasound focus was adjusted to facilitate the visualisation of deeper structures. The participants were positioned in a supine position with their heads maintained in a neutral position. Imaging was performed in both the transverse and longitudinal planes.

- Volume measurement

**Figure 1:**
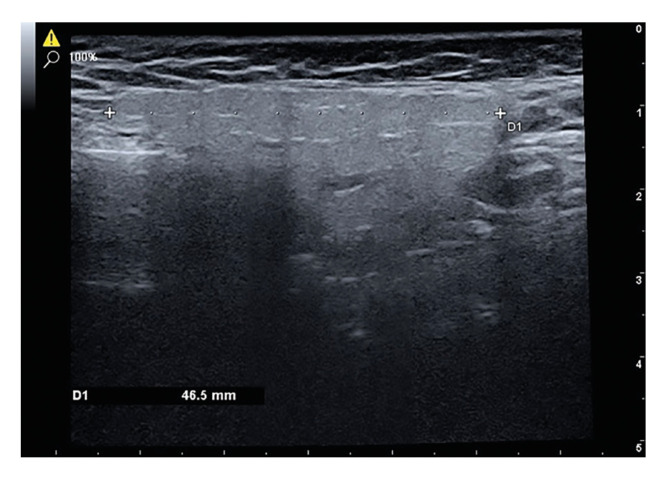
Superioinferior dimension measurement in a longitudinal ultrasonographic image of the parotid gland obtained using the panoramic scanning feature.

**Figure 2:**
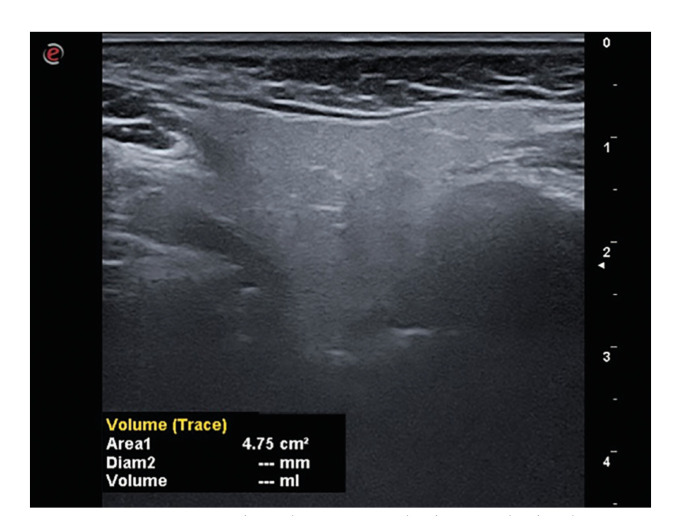
Transverse section ultrasonography image obtained to measure the volume of the parotid gland.

As the superio-inferior dimension of the gland could not be obtained on a single slice, a panoramic scan was performed in B-mode using the V-pan feature of the ultrasound device. The L4-15 probe was placed longitudinally on the parotid gland and scanned in the superio-inferior direction until the gland entered the image in all dimensions. When the desired image was obtained, it was frozen and the superio-inferior dimension of the gland was measured and recorded on the PACS system (Fig. 1). The probe was placed perpendicular to the ramus to obtain images in the transverse plane. When the gland boundaries were clearly visible, the image was frozen, the trace volume function of the device was selected and the gland boundaries were marked with the trackball. The device automatically calculated the volume by combining the dimension obtained in the longitudinal plane with the area value obtained by drawing the gland borders in the transverse plane (Fig. 2).

- Stiffness measurement

**Figure 3:**
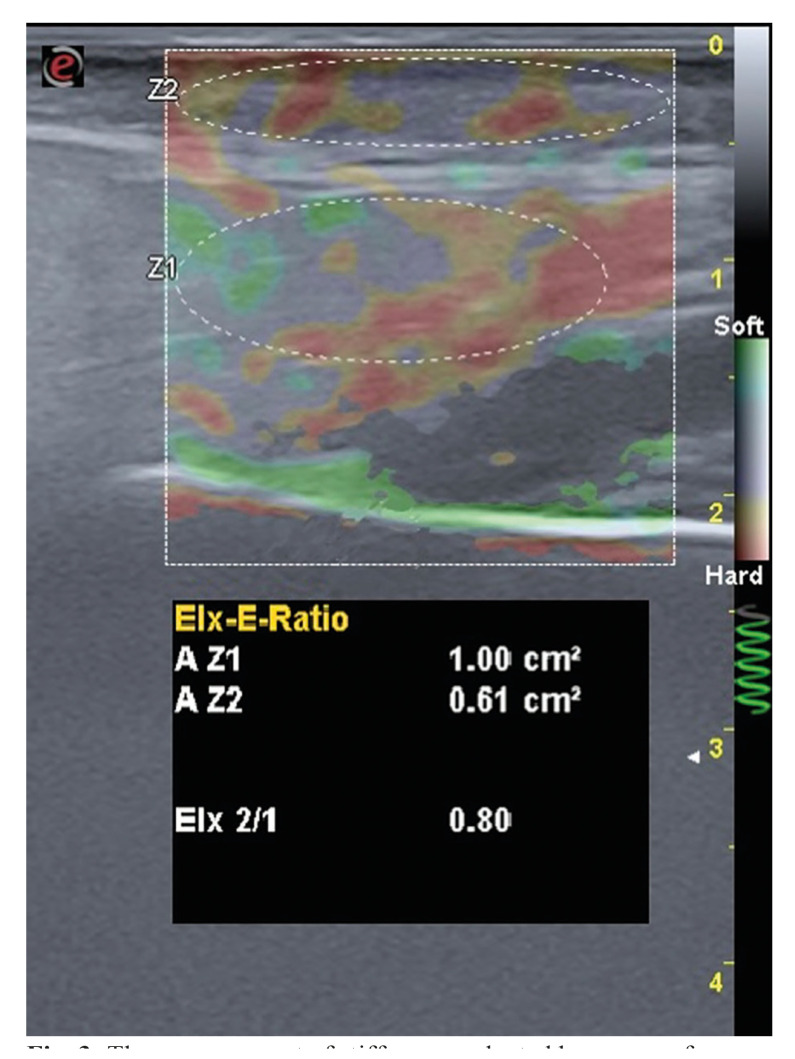
The measurement of stiffness conducted by means of a comparison with subcutaneous adipose tissue.

A strain elastography examination was conducted using the same device. ElaXto software on the ultrasound device was used to calculate the elasticity index (EI). Stiffness of the parotid gland was calculated as the ratio of the mean EI of the parotid gland to the mean EI of the subcutaneous adipose tissue. Region of interest (ROI)s were selected to encompass as much of the parotid gland and subcutaneous adipose tissue as possible (Fig. 3). The ROI was meticulously selected to exclude major vascular structures and lymph nodes. The optimum compression pressure was applied by following the elastic scale on the screen.

- Microvascularity measurement

**Figure 4:**
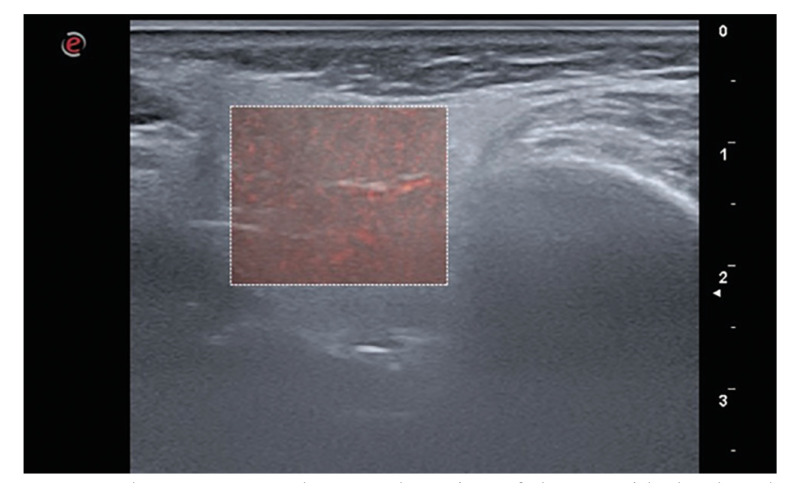
The transverse ultrasound section of the parotid gland, red coloured areas indicating the presence of microvessels and associated perfusion.

The ultrasound device's microV software was used to visualise the slow flow of microvessels in the parotid gland. Prior to microV imaging, participants rested for 10 minutes and were asked not to speak, breathe or swallow during imaging to eliminate motion artefacts. The ROI was set to the maximum size according to the anatomy of the glandular tissue in the transverse slices. The ROI was carefully selected to exclude major vascular structures and lymph nodes. The probe was held on the tissue until motion artefacts disappeared, and when the image was obtained, it was frozen and recorded on the unit's PACS system (Fig. 4). The recorded data were exported to a computer and analysed using PYTHON software. To measure microvascularity, the velocity index (VI), defined as the number of coloured pixels associated with the blood flow signal in the ROI/total number of pixels in the ROI × 100 (%), was calculated for both parotid glands.

- Statistical analysis

IBM SPSS 22.0 (Statistical Package for the Social Sciences) was used for data analysis. In the statistical analysis phase, descriptive statistics were first presented on the demographic characteristics of the participants. The Shapiro-Wilk and Kolmogorov-Smirnov normality tests were used to determine whether the data were normally distributed. Independent samples t-test was used for two-category variables with normal distribution, while Mann-Whitney U test was used for two-category variables without normal distribution. Pearson Product Moment Correlation Coefficient was used to determine the relationship between quantitative variables with normal distribution, while Spearman Rank Correlation Coefficient was used to determine the relationship between quantitative variables without normal distribution. All statistical tests used were tested at a 5% significance level.

## Results

The diabetes group comprised 24 females and 14 males, while the control group consisted of 29 females and 9 males. The mean age of the participants was 52.05 ± 13.94 years. The mean age of the diabetic group was 58.21 ± 11.86 years and the mean age of the control group was 48.89 ± 13.24 years.

Diabetic and control groups were compared in terms of volume, stiffness and microvascularity values of the parotid gland and the results are given in Table 1. It was determined that right volume, left volume, right stiffness and total volume values were significantly higher in the diabetic group compared to the control group (*p*<0.05). Left microvascularity and total microvascularity values were significantly higher in the control group than in the diabetic group (*p*<0.05).

In the diabetes group, insulin users and non-users were compared in terms of volume, stiffness and microvascularity values and the results are given in Table 2. No statistically significant difference was observed (*p*>0.05).

In the diabetes group, participants using antidiabetic agents and non-users were compared in terms of volume, stiffness and microvascularity values and the results are given in Table 2. Right stiffness and total volume values of the participants using antidiabetic agents were statistically significantly higher (*p*<0.05).

The relationship between HbA1c value and volume, stiffness and microvascularity values was investigated and the results are given in Table 3. No statistically significant relationship was found (*p*>0.05).

The relationship between the duration of exposure to diabetes and microvascularity was investigated, and the results are presented in Table 4. It was determined that there was a statistically significant negative and weak correlation between the duration of exposure to diabetes and left and total microvascularity (*p* < 0.05).

Dıscussıon

Investigation of parotid gland volume is necessary to establish reference values and to observe relationships between common diseases known to affect the parotid gland, such as DM and arterial hypertension. A various of studies have employed a variety of imaging modalities, including computed tomography (CT), positron emission tomography (PET-CT) and magnetic resonance imaging (MRI), to investigate parotid gland volume. The sample sizes of these studies range from 16 to 240. In these studies, the mean parotid volume was found to vary between 17 and 40 cm³ (12-17). In a recent cohort study by Brzoska *et al* (18). in which parotid gland volume was measured on non-contrast MRI images of 1725 participants, the mean volume was reported as 27.82 cm3 in males and 21.60 cm3 in females. In the present study, the volume values of the unilateral parotid gland were 41.29 ± 9.26 cm3 in the diabetic group and 31.88 ± 8.85 cm3 in the control group.

The most commonly cited causes of sialadenosis are chronic alcoholism and DM (19). The occurrence of painless parotid gland enlargement in obese and non-obese diabetic patients has been documented since the early 1900s. In a report published in 1981, Russotto observed that 24% of 200 patients with diabetes exhibited asymptomatic enlargement of the parotid glands. It should be noted, however, that this report was based on an inspection and palpation examination (20). There is a paucity of radiological studies investigating the relationship between diabetes mellitus (DM) and parotid gland size. Badarinza *et al* (21). ultrasonographically examined the parotid, submandibular and lacrimal glands of healthy and diabetic and/or obese patients in a prospective ultrasonographic study of 170 participants. The authors reported that the surface area of the parotid gland was found to be statistically significantly larger in the diabetic group compared to the control group. Gupta *et al*. (22) reported that parotid gland sizes were higher in diabetics according to the results of ultrasonography studies consisting of 100 diabetic and non-diabetic participants. Ozturk *et al*. (23) conducted an ultrasonographic examination of 80 participants, comprising diabetic and healthy subjects, and reported a significant increase in parotid gland size in the DM group. In the present study, similar to the studies of Badarinza, Gupta and Ozturk, it was concluded that the parotid gland volume was significantly higher in the diabetes group than in the control group.

HbA1c, or glycated hemoglobin, is a crucial marker in the management of diabetes. It serves as an indicator of long-term glycemic control and is a reliable predictor of diabetes-related complications (6). Therefore, the relationship between HbA1c level and parotid gland dimensions has been the subject of various studies. Gupta *et al* (22). observed a low-to-moderate positive correlation between HbA1c level and ultrasonographic dimensions of the parotid gland in diabetic patients. The results of the cohort MRI study conducted by Brzoska *et al*. (18), indicated a positive association between parotid gland volume in females and several anthropometric and biochemical parameters, including body mass index, waist circumference, serum triglyceride levels, and plasma HbA1c levels. It was proposed that the noTable correlation between HbA1c levels and gland volume in males may be more pronounced in a larger cohort. Badarinza *et al*. (21) did not identify a correlation between HbA1c and ultrasound parameters in diabetic patients. Similarly, no statistically significant correlation was observed between HbA1c levels and ultrasound parameters, including volume, stiffness and microvascularity, in this study.

Ultrasound imaging is a cruical tool in the evaluation of the salivary glands due to its efficiency, accessibility and non-ionising nature. The superficial location of the salivary glands makes ultrasound a suiTable tool for the initial assessment of these glands (24). Ultrasound elastography is a valuable tool for the evaluation of various parotid gland pathologies. It provides information on tissue elasticity and is useful for differentiating between benign and malignant lesions. The technique facilitates non-invasive evaluation and lesion characterisation by providing information about tissue elasticity and stiffness in diverse conditions, including parotid tumors (25). In their study comparing healthy, diabetic and/or obese individuals, Badarinza *et al* (21). evaluated the subjects with SWE and reported that there was no significant difference between the groups in terms of elastic modulus. Ozturk *et al* (23). reported that the SWE value was higher in the diabetic group than in the control group, although this finding was not statistically significant. A comparison with our study is challenging due to significant methodological discrepancies observed in published studies. These include the utilisation of disparate elastography techniques and probes with varying frequencies. In the present study, the strain elastography technique was employed, and no significant difference was identified between the diabetic and control groups in terms of stiffness. This finding corroborates the results of previous studies that have employed the SWE technique. It is well documented that patients with diabetes present with elastographic modifications of the liver associated with non-alcoholic liver disease. However, the results of studies, including the present study, indicate that there is no difference between the stiffness of the parotid glands of diabetic and non-diabetic participants. Badarinza *et al*. (21) proposed that a change in parotid gland stiffness is most likely related to a disease of the gland itself, rather than a response to metabolic syndrome. The authors also support this hypothesis.

Color Doppler imaging and microvascular imaging are both ultrasound techniques used to assess blood flow in tissues, but they have differences in their applications and capabilities. Color Doppler imaging is a widely used technique that visualizes blood flow direction and velocity in larger vessels by color-coding the information on a grayscale ultrasound image. It is effective for detecting fast flows in major vessels and provides information on blood flow patterns and abnormalities. On the other hand, microvascular imaging is a more advanced Doppler technology designed to detect slow blood flow in microvessels (26). Microvascular imaging is particularly useful for visualizing perfusion in small vessels with diameters of a few hundred micrometers, providing better qualitative and quantitative analysis of microvascular perfusion compared to color Doppler (27). Studies have shown that microvascular imaging techniques can reduce the influence of motion clutter, improve the detection rate of low-speed blood flow signals, and display tiny vessels more effectively than traditional color Doppler imaging (26). Long-term DM has been associated with decreased microvascular density and reduced vascular perfusion (28). Diabetes mellitus significantly affects microvascular flow, leading to dysfunction and impaired perfusion in various tissues and organs. Detection and management of microvascular changes in individuals with DM are critical in preventing complications (29). A review of the literature reveals a dearth of studies examining the vascularisation of the parotid gland in diabetic patients. In the limited number of existing studies, the colour Doppler mode was employed for the purpose of imaging. In their study, Badarinza *et al* (21). examined the parotid glands of participants with and without diabetes and/or obesity using colour Doppler ultrasound. The results demonstrated no statistically significant differences in vascularisation between the two groups. The findings of our study demonstrate a statistically significant decline in microvascularity within the diabetic group when compared to the control group. Additionally, a statistically significant negative correlation was identified between the duration of diabetes exposure and microvascularity. To the best of our knowledge, this is the inaugural study employing microvascular imaging to examine the parotid gland.

In conclusion, the study demonstrated a statistically significant increase in parotid gland volume and a reduction in microvascularity in diabetic patients. A statistically significant increase in parotid gland volume was observed in diabetic individuals who were using antidiabetic drugs in comparison to non-users. Furthermore, a significant negative correlation was identified between the duration of exposure to diabetes and microvascularity. The results of our study are of importance in terms of detecting decreased microvascularity and increased volüme in diabetics by ultrasound. However, there are some limitations to the as ultrasound is regarded as an operator-dependent imaging modality, it would be beneficial to compare the findings with those obtained from a different imaging modality, such as MRI. Furthermore, conducting a longitudinal follow-up of healthy subjects to examine the potential development of diabetes and a similar follow-up of diabetic subjects to investigate the possible progression of diabetes would enhance our understanding of the changes caused by diabetes in the parotid gland.

## Figures and Tables

**Table 1:** Comparison of volume, stiffness and microvascularity values according to groups.

Characteristics	Diabetes Mellitus	Healthy	Test St.	*p-value*
Right volume	39,65 ± 8,90	31,02 ± 8,99	t=4,202	0,000*
Left volume	42,93 ± 9,43	32,74 ± 8,74	t=4,882	0,000*
Total volume	41,29 ± 9,26	31,88 ± 8,85	t=6,401	0,000*
Right stiffness	0,51 ± 0,23	0,45 ± 0,35	U=532,0	0,049*
Left stiffness	0,62 ± 0,29	0,61 ± 0,28	t=0,178	0,860
Total stiffness	0,57 ± 0,27	0,53 ± 0,32	U=2483,0	0,136
Right MV	2,39 ± 2,04	2,49 ± 1,35	U=823,0	0,294
Left MV	1,46 ± 1,41	2,19 ± 1,42	U=971,0	0,010*
Total MV	1,93 ± 1,80	2,34 ± 1,38	U=3591,0	0,010*

*p<0,05; Test St.: test statistic; t: t test statistic; U: Mann-Whitney U test statistic; MV: microvascularity; +: Participants diagnosed with diabetes who use insulin or antidiabetic drug; -: Participants diagnosed with diabetes who do not use insulin or antidiabetic drug.

**Table 2:** Comparison of volume, stiffness and microvascularity values according to insulin and antidiabetic drug use in the diabetes mellitus group.

Characteristics	Insulin +	Insulin -	Test St.	P-value	Antidiabetic +	Antidiabetic -	Test St.	*P-value*
Right volume	40,69 ± 10,08	39,33 ± 8,68	t=0,395	0,695	40,92 ± 8,45	36,10 ± 9,62	t=1,494	0,144
Left volume	42,42 ± 8,35	43,09 ± 9,87	t=-0,183	0,856	44,14 ± 8,65	39,54 ± 11,11	t=1,336	0,190
Total volume	41,55 ± 9,03	41,21 ± 9,40	t=0,138	0,891	42,53 ± 8,63	37,82 ± 10,27	U=372,0	0,027*
Right stiffness	0,43 ± 0,18	0,54 ± 0,23	t=-1,292	0,205	0,57 ± 0,21	0,37 ± 0,20	t=2,516	0,016*
Left stiffness	0,70 ± 0,51	0,60 ± 0,19	t=0,911	0,571	0,58 ± 0,22	0,74 ± 0,44	t=-1,408	0,168
Total stiffness	0,57 ± 0,40	0,57 ± 0,21	U=602,5	0,325	0,57 ± 0,21	0,55 ± 0,38	U=464,5	0,260
Right MV	2,58 ± 1,21	2,33 ± 2,26	U=97,5	0,262	2,41 ± 2,04	2,33 ± 2,16	U=129,5	0,732
Left MV	1,71 ± 1,03	1,39 ± 1,51	U=93,5	0,208	1,56 ± 1,50	1,20 ± 1,11	U=118,5	0,482
Total MV	2,14 ± 1,18	1,86 ± 1,96	U=382,5	0,088	1,98 ± 1,83	1,76 ± 1,77	U=490,5	0,412

*p<0,05; Test St.: test statistic; t: t test statistic; U: Mann-Whitney U test statistic; MV: microvascularity; +: Participants diagnosed with diabetes who use insulin or antidiabetic drug; -: Participants diagnosed with diabetes who do not use insulin or antidiabetic drug.

**Table 3:** The relationship between HbA1c and volume, stiffness and microvascularity in the diabetes mellitus group.

Characteristics	HbA1c
Volume (total)	-0,083 (0,484)
Stiffness (total)	-0,154 (0,189)
Microvascularity (total)	-0,109 (0,354)

*p-value<0,05.

**Table 4:** Association of microvascularity with duration of exposure to diabetes in the diabetes mellitus group.

Microvascularity	Duration of exposure to DM
Right	-0,250 (0,129)
Left	-0,382* (0,018)
Total	-0,301* (0,008)

*p<0,05; DM: Diabetes mellitus.
